# PFCs and Early Menopause: Association Raises Questions about Causality

**DOI:** 10.1289/ehp.122-A59

**Published:** 2014-02-01

**Authors:** Lindsey Konkel

**Affiliations:** Lindsey Konkel is a Worcester, MA–based journalist who reports on science, health, and the environment. She is an editor for *Environmental Health News* and *The Daily Climate*.

Polyfluoroalkyl chemicals (PFCs)—chemicals used in the manufacture of stain- and water-repellant products—are suspected endocrine disrupters.^1^ Animal studies have suggested an association between exposure to two of the most commonly encountered PFCs (perfluorooctanoate, or PFOA, and perfluorooctane sulfonate, or PFOS) and altered hormonal function,^1^^,^^2^ although evidence in humans remains inconsistent. A small number of cross-sectional epidemiological studies have reported an association between PFOA/PFOS exposure and timing of puberty,^3^ menopause,^4^ and thyroid function,^5^ while others have reported no association.^6^ In this issue of *EHP*, researchers report an association between PFC concentrations in circulating blood and rate of hysterectomy as well as earlier age at natural menopause.^7^

The findings, say the researchers, raise the question of whether high PFC exposure may lead to early menopause or, conversely, whether early menopause may lead PFCs to build up in women’s bodies. Either way, the results are of interest because women who reach menopause by their late 40s are at increased risk for various forms of heart disease.^8^^,^^9^^,^^10^ “If PFC levels are predictors of earlier menopause,” the investigators write, “exposure may also increase the risk of other serious health outcomes (e.g., cardiovascular disease and stroke).”^7^

According to the North American Menopause Society, menopause occurs, on average, at age 51 in U.S. women. Women characterized as having early or premature menopause will have had their last menstrual period before age 40.^11^

In the current study, researchers from the National Institute of Environmental Health Sciences (NIEHS) and the University of North Carolina at Chapel Hill analyzed data for 2,732 women, more than two-thirds of whom were premenopausal. The investigators compared blood concentrations of four PFCs—PFOA, PFOS, perfluorononanoate (PFNA), and perfluorohexane sulfonate (PFHxS)—and, for postmenopausal women, age at natural or surgically induced menopause. The data were collected as part of the National Health and Nutrition Examination Survey (NHANES) between 1999 and 2010.

NHANES data are intended to be nationally representative, and past analyses have indicated ubiquitous exposure to PFOS, PFOA, and other PFCs. One 2007 NHANES analysis showed that 98% of adults sampled had measurable levels of at least two PFCs in their blood.^12^

In the current study, women with the highest blood levels of PFOA, PFNA, and PFHxS were, respectively, 36%, 47%, and 70% more likely to have experienced menopause than women with the lowest concentrations of these compounds. Premenopausal women had the lowest levels of all four PFCs, whereas women who had undergone hysterectomy had the highest levels.^7^

PFHxS was most strongly associated with rate of hysterectomy. Women with the highest blood levels were 3.5 times more likely to have had a hysterectomy than women with the lowest levels. PFOA, PFNA, and PFOS were also significantly associated with having had a hysterectomy.^7^

**Figure:**
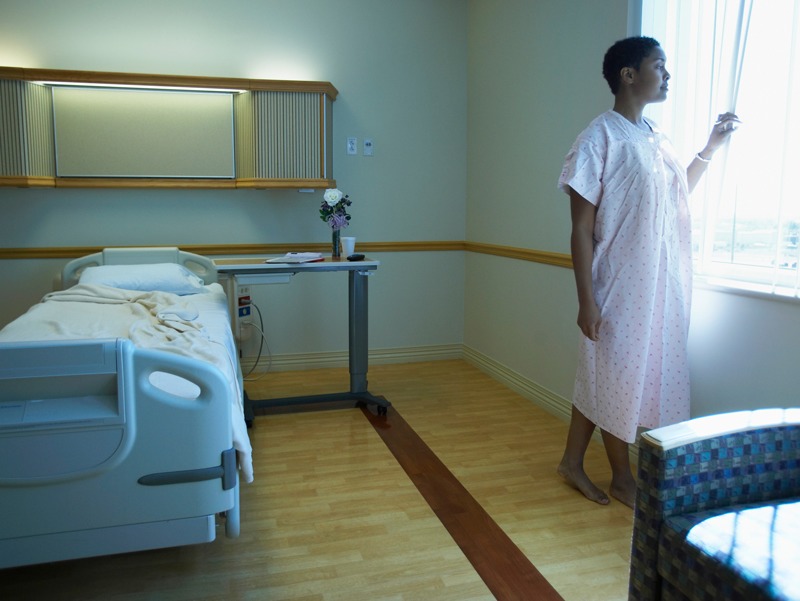
Hysterectomy was associated with the highest blood levels of all four PFCs studied, possibly a result of unusually early menopause— although longitudinal studies are necessary to test that hypothesis. © Tim Pannell/Corbis

Women in the study reached natural menopause at a median age of 49, while women who had hysterectomies reported their last period at a median age of 38. The cross-sectional nature of the study makes it impossible to distinguish whether higher PFC levels may have led to earlier menopause or vice versa.

“PFCs circulate in blood, so menstruating women may lose PFCs through blood loss, which would not be the case for postmenopausal women,” says lead author Kyla Taylor, a health scientist at the NIEHS. Whereas many persistent organic pollutants are stored primarily in fat tissue, PFCs form bonds with blood proteins. That means they tend to accumulate in serum rather than in fat.

“While this study suggests the potential for reverse causation in the association between PFCs and earlier menopause, we don’t know what that means or whether it’s the sole explanation [for the association],” says Sarah Knox, an epidemiologist at West Virginia University who studies PFCs but was not involved in this research. Only a longitudinal study can provide the insight to determine causality. “This is an area of concern and an area clearly in need of further research,” Knox says.
